# Supplementary material for: The geometric evolution of aortic dissections: Predicting surgical success using fluctuations in integrated Gaussian curvature
Source: PLoS Comput Biol. 2024 Feb 2;20(2):e1011815. doi: 10.1371/journal.pcbi.1011815 (PMC10866512; doi:10.1371/journal.pcbi.1011815)
Supplement: S1 Appendix — Fig A includes the demographic information for the non-pathologic aortic cohort. Fig B is the demographic information for the dissection cohort. Section titled “Aortic Segmentation and Post-Processing from CTA Imaging” includes details on the methods and procedures involved in Segmentation, Noise Reduction, Smoothing, Isolation of the Outer Surface of the aortic mask, and Meshing. The section on “Calculation of the Shape Operator” details our implementation of the Rusinkiewicz algorithm of calculating surface curvatures on a meshed surface which are the primary inputs into our shape and size calculations. The section “Artifact Removal” details the criteria used to remove the flat edges and and rims which are generated during the segmentations and constitute artifacts. The section “Jensen-Shannon Divergence of Partition Gaussian Curvature” details our implementation of the JSD as a measure of κg spatial gradients within partitions. The section titled “Sensitivity to Partition Size” details our exploration of how patch size impacts the distribution of data projected into the shape-size feature space. The section “Ideal Shapes” provides the analytical functions used to generate the idea shapes used for cross-validation of our methods in the manuscript. The section “Other Shape Metrics” shows our detailed exploration of other published functions quantifying shape and the projection of our data into each one of the individual shape-size feature spaces. The section “Finite Element Simulations” provides details of material model selection and element selection for the FEA simulations in the paper. And the final supplementary section “Analysis on Pre-Operative Data” projects only the last pre-operative scan into the shape-size feature space, this is a reduced dataset of the full data set provided in Fig 7 of the paper. (PDF) [file pcbi.1011815.s001.pdf]

Supplemental Information: The Geometric Evolution of Aortic Dissections: Predicting Surgical Success using Fluctuations in Integrated Gaussian Curvature

Kameel Khabaz[1], Karen Yuan[1], Joseph Pugar[1,2], David Jiang[1], Seth Sankary[1], Sanjeev Dhara[1], Junsung Kim[1], Janet Kang[1], Nhung Nguyen[1], Kathleen Cao[1], Newell Washburn[3], Nicole Bohr[1], Cheong Jun Lee[4], Gordon Kindlmann[5], Ross Milner[1], Luka Pocivavsek[1*]

1 Department of Surgery, The University of Chicago, Chicago, Illinois, United States of America
2 Departments of Material Science and Engineering, Biomedical Engineering, and Chemistry, Carnegie Mellon University, Pittsburgh, Pennsylvania, United States of America
3 Department of Biomedical Engineering, Carnegie Mellon University, Pittsburgh, Pennsylvania, United States of America
4 Department of Surgery, NorthShore University Health System, Evanston, Illinois, United States of America
5 Department of Computer Science, The University of Chicago, Chicago, Illinois, United States of America

* lpocivavsek@bsd.uchicago.edu

Demographic Information

Demographics (N = 93)	N	%		
Male	66	71		
Female	27	29		
White	9	5.6		
Declined	2	5.6		
Black	75	83		
Other	7	5.6		
	Mean	SD	Min	Max
Age	37.08	18.10	1	86
Comorbidities	N	%		
Diabetes	6	6.4		
Hypertension	11	11.8		
COPD	5	5.4		
Smoking	9	9.7		
Coronary Artery Disease (CAD)	2	2.2		
Peripheral Artery Disease (PAD)	0	0		
Heart Failure	3	3.2		
Stroke	0	0		

Fig A. Non-Pathologic Aortic Cohort Patient Information

Demographics (N = 36)	N	%		
Male	21	58		
Female	15	42		
White	9	25		
Declined	2	5.6		
Black	22	61		
Other	3	8.3		
	Mean	SD	Min	Max
Age (Successful TEVAR)	59.06	10.88	36	82
Age (Failed TEVAR)	67.44	15.52	39	93
Comorbidities	N	%		
Hypertension	32	94		
Known Marfan	0	0		
COPD	7	21		
Smoking	24	71		
CAD	11	32		
PAD	4	12		
Heart Failure	9	26		
Diabetes	4	12		
Stroke	4	12		
Acute	3	8.8		
Complicated	3	8.3		
Device Information	Mean	SD	min	max
# Devices	2.08	1.20	1	6
# Reinterventions	1.33			
Proximal Oversizing	20%	0.104%	2%	48%
	N	%		
Z3 Landing Zone	21	58		
Z2 Landing Zone	12	33		
Z1 Landing Zone	2	6		
Z0 Landing Zone	1	3		
Medtronic Navion Device	25	69		
Gore TAG Device	10	28		
Cook TX2 Device	1	3		
Reintervention	15	42		
Endoleak	16	50		
Imaging Data	Mean	SD	min	max
Follow Up Time (Years)	1.78	1.69	0.074	5.85
Total # CT	6.81	2.91	3	15
Time to Intervention	1.33	2.36	0	9.61
Post-op # CT Scans	4	2.26	1	11

Fig B. Type B Aortic Dissection Cohort Patient Information

Aortic Segmentation and Post-Processing from CTA Imaging

Segmentation

Segmentation of the aorta is performed using a custom semi-automated workflow built to characterize global aortic geometry. The process first uses a threshold filling function to select voxels based on greyscale values. The threshold is then manually corrected by examining each axial slice, erasing non-aortic tissue, and performing other corrections as needed. The outer edge of the segmentation mask corresponds to the aortic inner wall, which is the most robust method of selecting the aortic surface given the difficulty in correctly delineating the outer aortic wall on CTA. The segmentation length is cut at the aortic sinus, the branching of the brachiocephalic artery, and at the celiac artery. As the goal is to analyze aortic geometry, branch vessels are removed from the segmentation using a curved selection with a 3D edit tool. Segmentation accuracy is independently reviewed by the senior author.

Noise Reduction

Segmentation of accurate patient-specific geometries from real CTA imaging data comes with several sources of noise, including inconsistent imaging quality and/or resolution, imaging artifacts, non-contrast-enhanced tissues like aortic thrombus, or residual variability between segmentations. Therefore, noise reduction is a key step of the workflow and includes the application of a thorough segmentation smoothing algorithm and a robust meshing algorithm. For each algorithm, key parameters are varied so that 15 distinct models are created for the one segmentation. This significantly reduces segmentation noise, as the models are aggregated later in the geometric analysis workflow.

Smoothing

The smoothing algorithm is a five-step process optimized to decrease noise while preserving local geometric features. It consists of dilation of the segmented geometry, the application of a mean filter, the application of a median filter, the application of a recursive Gaussian filter, and erosion. The segmentation is dilated (and later eroded) by four pixels. The mean and median filters compute each pixel's value as a statistical mean and median of neighboring greyscale values within a radius of five pixels. The recursive Gaussian filter applies a smooth blur to the segmentation mask. Three models with three different widths of Gaussian kernels are made: a standard deviation of six, seven, and eight pixels in each direction for segmentations of dissections and four, five, and six pixels for segmentations of non-pathologic aortas. These parameters are the result of a thorough parameter sweep, in which the effect of changing each parameter on the resulting curvature values was analyzed.

Isolation of the Outer Surface

As aortas are systems of highly varying curvatures, a constant orientation of the normal vectors is maintained by isolating of the outer surface of the segmentation. The outer surface is isolated by making a closed surface of the smoothed segmentation, corresponding to the outer edge of the aortic inner wall, with the longitudinal ends defined by planes. The intersection of the planes defining the end of the segmentation and the shell creates rims of sharp surface curvature that are removed later in the workflow.

Meshing

A triangular mesh for the outer surface is generated for each smoothed segmentation in ScanIP by creating a Matlab (2021b, Mathworks, Natick, MA) surface model with a minimum feature size of 0.5 mm and at 5 separate dimensionless coarseness parameters (on a scale from −50, very coarse, to 50, very fine, according to the program's internal scaling): −35, −30, −25, −20, −15. This correlates to a mesh density of 0.5 elements/mm^2 to 1.5 elements/mm^2. These parameters represent optimal mesh densities and were found by studying the effect of changing the mesh density on calculated curvature values for analytically ideal shapes (e.g. a sphere, torus, cylinder). ScanIP uses a robust meshing algorithm, called "+FE Free," to create accurate surface meshes for complex surface geometries. First, the algorithm generates a "+FE Grid" high-quality mesh characterized by constant element edge lengths. Then, the surface is remeshed with the "+FE Free" algorithm with adaptive surface remeshing to enhance the mesh quality.

Therefore, for each segmentation, the 15 different outer surface meshes are created and analyzed as a result of the 3 smoothing parameter variants and 5 meshing parameter variants. This controls for the known variability of discrete derivatives calculated on meshed surfaces, and the variability generated from the noise reduction procedure is indicated by the error bars in the main paper Figs 7, 11, and 12 [1].

Calculation of the Shape Operator

The Rusinkiewicz algorithm of estimating surface curvature computes the derivatives of surface normals [1] in order to calculate the per-vertex shape operator for each mesh. This algorithm excels at handling irregular triangular surface meshes, efficiently performing computations, and providing robust results on local surface neighborhoods. Briefly, the algorithm calculates the per-vertex shape operator as a weighted average of the shape operators of immediately adjacent faces. The per-face tensors are computed using a finite-difference approximation defined in terms of the directional derivative of the surface normal [1]. The algorithm was implemented in MATLAB [2].

First, the per-face normal vectors are calculated as $\vec{n}_m = \vec{e}_1 \times \vec{e}_2$, in which $\vec{e}_1$ and $\vec{e}_2$ are two unit-length edges of the triangular surface mesh. The per-vertex normal vectors $\vec{n}_i$ are then calculated using a weighting algorithm to accurately estimate vertex normals [3]. For each face m of area A_m containing three edges ($\vec{e}_0$,$\vec{e}_1$,$\vec{e}_2$), each of the 3 vertices is assigned a weight: $w_{m,1} = \frac{A_m}{|\vec{e}_1|^2|\vec{e}_2|^2}$, $w_{m,2} = \frac{A_m}{|\vec{e}_0|^2|\vec{e}_2|^2}$, $w_{m,3} = \frac{A_m}{|\vec{e}_1|^2|\vec{e}_0|^2}$. Then, the contribution of face m to the normal vector of vertex i is $w_{m,i}\vec{n}_m$, which is added to $\vec{n}_i$. After this is done for all faces containing vertex i, the per-vertex normal vectors are normalized to unit length [1].

A per-vertex $(\vec{u}_i, \vec{v}_i)$ coordinate system is calculated in which $\vec{u}_i = \vec{e}_m \times \vec{n}_i$ and $\vec{v}_i = \vec{n}_i \times \vec{u}_i$. $\vec{e}_m$ indicates a face edge vector touching vertex i. This per-vertex coordinate system will later be utilized for averaging the per-face shape operator with the contributions of adjacent faces to calculate the per-vertex tensor [1].

Next, the per-face shape operator is calculated. For each face m, a $(\vec{u}_m, \vec{v}_m)$ coordinate system is defined as $\vec{u}_m = \vec{e}_0$ and $\vec{v}_m = \vec{n}_m \times \vec{e}_0$. $\vec{n}_0$, $\vec{n}_1$, and $\vec{n}_2$ are defined as the normal vectors of the three vertices constituting the face. Then, the second fundamental form $\mathbb{I}$ is calculated by solving a least-squares problem:

$$\mathbb{I}\begin{pmatrix}\vec{e}_0 \cdot \vec{u}_m \\ \vec{e}_0 \cdot \vec{v}_m\end{pmatrix} = \begin{pmatrix}(\vec{n}_2 - \vec{n}_1) \cdot \vec{u}_m \\ (\vec{n}_2 - \vec{n}_1) \cdot \vec{v}_m\end{pmatrix} \tag{1}$$

$$\mathbb{I}\begin{pmatrix}\vec{e}_1 \cdot \vec{u}_m \\ \vec{e}_1 \cdot \vec{v}_m\end{pmatrix} = \begin{pmatrix}(\vec{n}_0 - \vec{n}_2) \cdot \vec{u}_m \\ (\vec{n}_0 - \vec{n}_2) \cdot \vec{v}_m\end{pmatrix} \tag{2}$$

$$\mathbb{I}\begin{pmatrix}\vec{e}_2\cdot\vec{u}_m\\ \vec{e}_2\cdot\vec{v}_m\end{pmatrix}=\begin{pmatrix}(\vec{n}_1-\vec{n}_0)\cdot\vec{u}_m\\ (\vec{n}_1-\vec{n}_0)\cdot\vec{v}_m\end{pmatrix} \tag{3}$$

The shape operator is then projected onto each of the face's vertices. To avoid a "loss" of curvature at the change from the vertex-based coordinates $(\vec{u}_i, \vec{v}_i)$ to the face-based coordinates $(\vec{u}_m, \vec{v}_m)$, one of the coordinate systems is rotated to be coplanar with the other, as previously performed in the literature [1]. Similarly to the calculation of the per-vertex normal vectors, the contribution of each touching face is weighted to form the per-vertex shape operator. In contrast to the weighing procedure for calculating the normal vectors, previous literature [1] utilizes a "Voronoi area" weighting procedure for finding the per-vertex shape operator, which is followed here. This calculates $\vec{w}_{m,i}$ as the portion of the area of face m that lies closest to vertex i. This approach has been found to produce the best estimates of curvature for faces of varying sizes and shapes [1]. This tensor is then diagonalized to obtain the per-vertex principal curvatures k_{1i}, k_{2i}. This results in the per-vertex principal curvatures for each aortic surface geometry's 15 meshes.

Artifact Removal

The isolation of the outer surface of the aortic segmentations, which is performed to maintain a consistent orientation of normal vectors, results in the creation of flat edges with rims of sharp curvature. The contribution of these flat edge regions is removed before calculating the curvature functions. As artifacts, these points have outlying curvature values. Flat edges are removed by removing vertices with $|k_{1i}| < |\frac{k_m}{2000}|$ and $|k_{2i}| < |\frac{k_m}{2000}|$, where k_m is the average of the per-vertex mean curvature $(\kappa_m = (k_{1i} + k_{2i})/2)$ for the entire surface. This threshold value was carefully chosen to be overly specific, and each segmentation is manually verified to confirm that only the flat edges are removed.

Removing the sharp rims required separate criteria for normal and diseased aortas. For the diseased aortas, points with $|\kappa_{gi}| > \overline{\kappa_{gi}} + 2\sigma_{\kappa_{gi}}$ and $|k_{2i}| > \overline{k_{2i}} + 3\sigma_{k_{2i}}$ are removed. Here, $\overline{\kappa_{gi}}$ indicates the mean per-vertex Gaussian curvature (κ_{gi}) of the aorta, and $\sigma_{\kappa_{gi}}$ indicates the standard deviation of the Gaussian curvature. Similarly, $\overline{k_{2i}}$ is the mean second principal curvature (k_{2i}), which is defined as the principal curvature with a larger absolute value. For normal aortas, points with $|\kappa_g| > \overline{\kappa_g} + 1.1\sigma_{\kappa_g}$ and $|k_{2i}| > \overline{k_{2i}} + 1.6\sigma_{k_{2i}}$ are removed. These parameters are manually chosen to remove outlying points without removing points actually on the outer surface. On average, around $1-2\%$ of mesh vertices are removed as part of the rims of sharp curvature. Representative examples of the results of both artifact removal procedures are shown in Fig C. For the ideal shapes, no points are removed for the sphere, points of positive Gaussian curvature are removed for the catenoid and pseudosphere, and the same procedure as for diseased aortas is utilized for the torus and cylinder, resulting in an average of 0.03% of points being removed.

Jensen-Shannon Divergence of Within-Partition Gaussian Curvature

The method requires that κ_{gi} is relatively constant inside each surface partition, such that $K_j \approx A_j\overline{\kappa}_{gj}$, where $\overline{\kappa}_{gj}$ is the mean Gaussian curvature within partition j. As discussed in the main text, the partitioning is implemented via a simple Voronoi

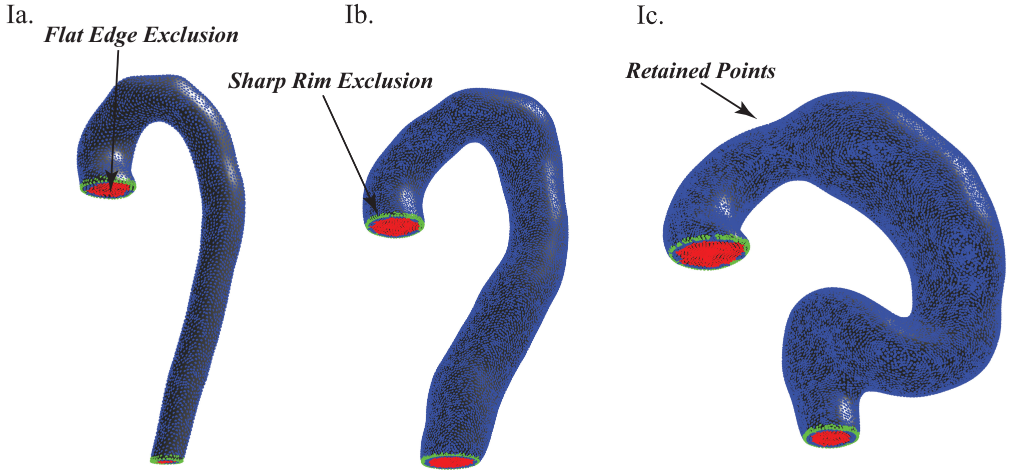

Fig C. Artifact Removal Demonstration of artifact removal procedures. Points excluded using the flat edge removal procedure are shown in red, and points excluded using the sharp rim removal procedure are shown in green. Retained points are shown in blue. Representative examples are shown for Ia. a non-pathologic aorta, Ib. a diseased aorta in the successful TEVAR cohort, and Ic. a diseased aorta in the failed TEVAR cohort. As can be seen, these artifact removal procedures are more specific than sensitive, in that not all artifact points have been removed, but very few (if any) non-artifact points are incorrectly excluded. Images are not drawn to scale.

decomposition without explicitly taking local curvatures into account. To calculate the degree of κ_{gi} variability within each partition for a given decomposition, the Jensen-Shannon Divergence (JSD) is used. The JSD is an information-theoretic measure of dissimilarity among probability distributions, and it is used here to quantify the discrepancy between the ideal partition of weakly varying κ_{gi} and the true surface partition [4].

First, an aortic surface with weakly varying κ_{gi} is defined as having a κ_{gi} distribution centered at $\overline{\kappa}_{gj}$, the mean Gaussian curvature in the partition, with a standard deviation of $\sigma = 0.1|\overline{\kappa}_{gj}|$. Although this definition is arbitrary, it precisely defines a tight distribution with minimal variation in κ_{gi} that appropriately scales with the magnitude of curvature in a partition. For distributions $f_1(X)$ and $f_2(X)$, $JSD = \frac{1}{2}(D(f_1(X)||\tilde{f}(X)) + D(f_2(X)||\tilde{f}(X)))$, where $\tilde{f}(x) = \frac{1}{2}(f_1(x) + f_2(x))$ is the common distribution and $D(g(X)||f(X)) = \int_{-\infty}^{\infty} g(x) \ln \frac{g(x)}{f(x)}\, dx$ is the relative entropy, which captures the divergence from the given probability distribution $f(X)$ to the reference distribution $g(X)$ [5,6]. The discrete form $D(g(X)||f(X)) = \sum_{x \in X} g(x) \ln \frac{g(x)}{f(x)}$ is calculated. The Freedman–Diaconis rule is used to optimize the calculation of the JSD by properly discretizing the κ_g distribution $f(X)$ for partition-level data $X = \{\kappa_{g_1}, \kappa_{g_2}, ..., \kappa_{g_n}\}$ into m bins, where $m = \frac{\sqrt[3]{n}(\max(X) - \min(X))}{2\,\mathrm{IQR}(x)}$ [7]. The reference distribution $g(X)$ is defined by randomly sampling 10,000 values from a Gaussian distribution with a mean of $\overline{\kappa}_{gj}$ and $\sigma = 0.1|\overline{\kappa}_{gj}|$ and then creating a histogram. Both distributions are normalized to probability mass functions prior to calculating the JSD of the partition.

Fig D visually demonstrates the calculation of the Jensen-Shannon divergence. On average, partitions have a JSD of under 0.2 (see dotted line in panel III), compared to a maximum bound of ln(2). This demonstrates that partitions have a relatively weakly varying κ_g when $k = A_T/\ell^{-2}$ partitions are defined. Fig D III also shows that the JSD increases for a coarser partitioning ($k/10$ partitions). In this limit, the method of

calculating K is no longer valid. The JSD is reduced for a higher density partitioning ($k * 10$ shown). This identifies a range of partition densities within which the extrinsic calculation of local K is valid.

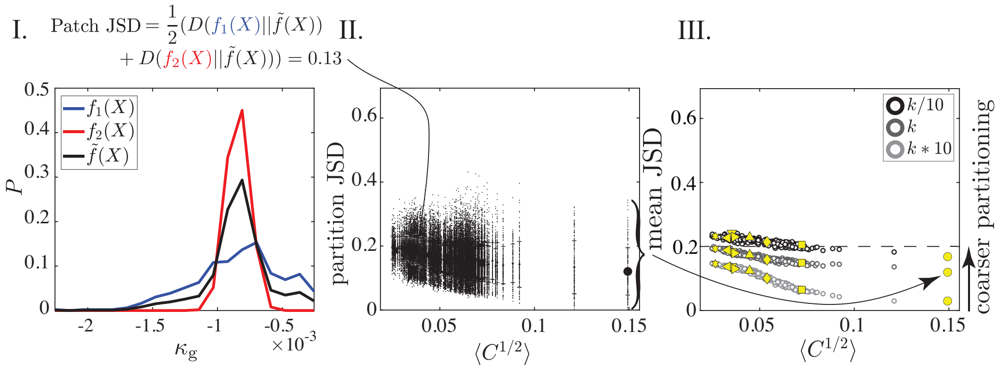

Fig D. Quantitating per Patch κ_g Gradients using Statistical Analysis Calculation of Jensen–Shannon divergence (JSD) to quantify the variability of within-partition Gaussian curvature. I. The JSD is calculated for a partition by comparing $f_1(X)$, the κ_{g} distribution, with $f_2(X)$, the reference distribution with weakly-varying κ_{g}. II. Plot of the JSD for all partitions versus $\langle C^{1/2} \rangle$ for the corresponding aorta. III. The mean JSD is calculated for all 302 aortas (yellow symbols correspond to the canonical 8 aortas, see Fig 1 of the main paper). The JSD is compared for three partitioning densities. At the base level of k, JSD < 0.2 out of a theoretical maximum of ln(2). When k increases by a factor of 10, the mean JSD decreases for all patients, indicating that the weakly varying curvature partition assumption becomes more valid. When k decreases by a factor of 10, the mean JSD increases for all patients, indicating that the weakly varying κ_{g} assumption is less valid.

Sensitivity to Partition Size

As detailed in the main text, each aorta is subdivided into $k = A_T \ell^{-2}$ partitions using a k-means algorithm. This achieves an approximately constant number of partitions, each sized according to a self-contained length scale, across all aortas. Fig D III shows that $JSD < 0.2$ and the extrinsic computation of k remains valid for a number of partitions greater than k. If the number of partitions decreases, JSD increases and the computation of K is invalid. In this section, the impact of partition number on the curvature functions δK and $\langle C^{1/2} \rangle$ is studied. The number of partitions is increased or decreased by a factor of 10. The calculations are also performed on the level of a single mesh element.

Fig E shows the data presented in Fig 7 III of the main paper with the calculations performed on different partition densities and with no partitioning at the level of the mesh. As outlined in the main text, δK captures the balance of positive and negative curvatures across the aortic surface in a globally scale-invariant manner. For aortas of different sizes but constant shape (due to normal growth during development), δK must be constant. Fig E IV shows that δK does not follow the trend when the computations are performed on the level of the mesh. We hypothesize that the breakdown of the computation in this limit occurs because the mesh is composed of flat Euclidean triangles. Diguet's Theorem states that for surface patches of constant κ_{g} (low JSD in our case), the surface area scales as $A_j \sim \pi(r_i^2 - \kappa_{\mathrm{g}_i} r_i^4)$ where r_i extends from the patch center along a geodesic line [8,9]. As said, for $r_i \sim \sqrt{a_m}$, the area calculation does not correctly account for the local curvature and one would need access to the metric to

perform the integration. The breakdown of the scaling with coarsened partitions, shown in Fig E I, is also discussed in Jensen-Shannon Divergence of Within-Partition Gaussian Curvature; $JSD > 0.2$ invalidates the ability to take κ_g outside the integral.

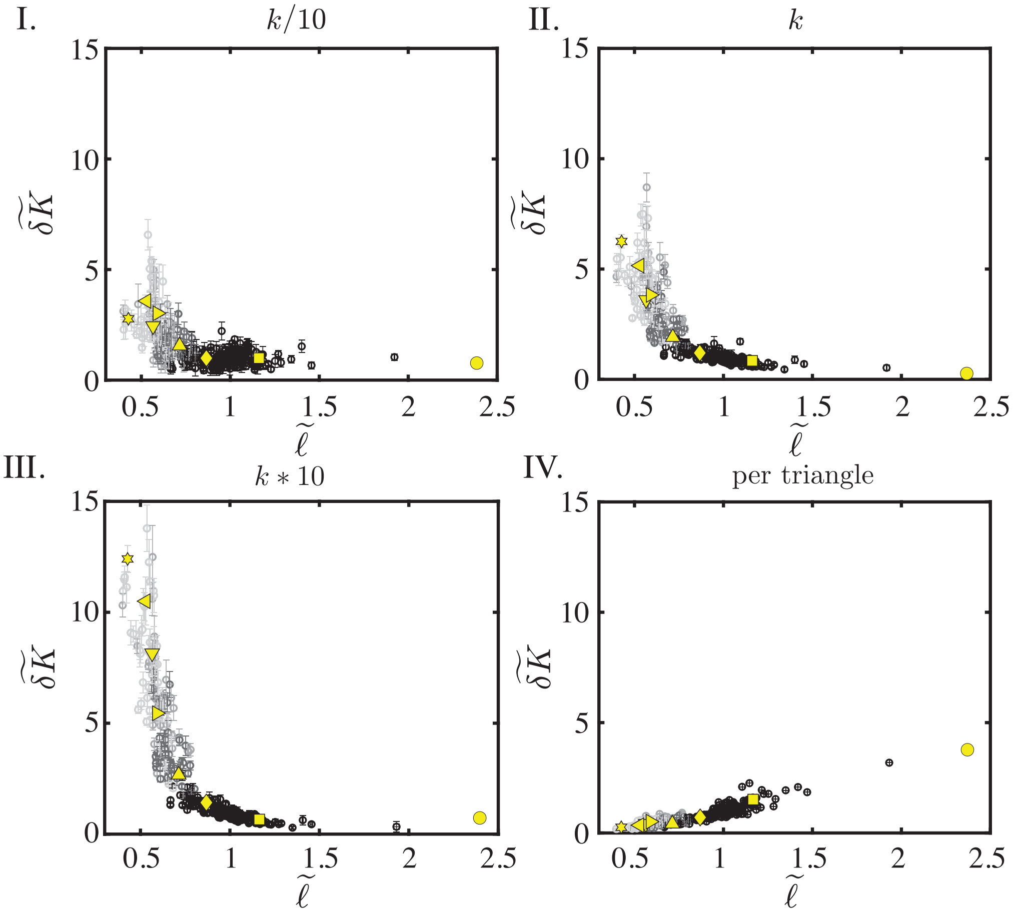

Fig E. Partitioning Sensitivity Analysis - Signal Loss at Coarse and Dense k Values Tuning the partition number k demonstrates the sensitivity of the method to partition size. The surface is partitioned into $k = A_T \ell^{-2}$ partitions to obtain A_j, the area over which the partition-level total curvature $K_j = \iint_{A_j} \kappa_{g_i} da_m$ is taken. This number remains on the order of $O(100)$ for many aortic shapes and sizes. I. When k is divided by 10, there is a clear increase in noise causing higher variation in δK for the normal aortas, poor separation of the three patient groups, and a suppressed divergence in δK for highly diseased aortas (light gray). II. The base level of k. III. Multiplying k by 10 (resulting in a larger partition number and smaller partition size) shows that the general feature signal contained in δK is retained. IV. Performing the calculation at a per-triangle level, at which $K_i = \kappa_{g_i} a_{mi}$ is the total curvature of one triangle, inverts the direction of the trend and degrades the signal. This is because individual element areas a_m are locally Euclidean and thus cannot individually approximate a curved surface.

Ideal Shapes

Before continuing with the geometric analysis workflow, the ideal shapes used in Fig 7 II and III in the main text are created to demonstrate that the sum of total curvature ($\sum K$) is a topologic invariant. Spheres, tori, cylinders, pseudospheres, and catenoids of varying sizes are defined using MATLAB. For each geometry, point clouds are defined from the respective functional form and triangulated into a surface mesh. Cylinders are created with radii $r = \{5, 7, 9, 14, 28, 35\}$ mm and heights $h = 2r$ for a total of 6 models. Spheres are defined with radii $r = \{7, 8, 9, 10, 13, 20, 25, 50\}$ mm to obtain 8 models. Tori are defined using the following parametric equations:

$$x = (c + a\cos v)\cos u \tag{4}$$
$$y = (c + a\cos v)\sin u \tag{5}$$
$$z = a\sin v \tag{6}$$

for $u, v \in [0, 2\pi)$, where $c = \{11, 14, 20, 35, 50, 60\}$ mm and $a = c/2$ (for 6 models).

Likewise, the following Cartesian parametric equations define the catenoids:

$$x = c\cosh(v/c)\cos u \tag{7}$$
$$y = c\cosh(v/c)\sin u \tag{8}$$
$$z = v \tag{9}$$

for $u \in [0, 2\pi)$, with $c = \{2, 8, 10\}$ mm and $v \in [-c, c]$.

The pseudosphere is similarly defined according to the Cartesian parametric equations

$$x = c\,\mathrm{sech}\,v\cos u \tag{10}$$
$$y = c\,\mathrm{sech}\,v\sin u \tag{11}$$
$$z = v - \tanh v \tag{12}$$

for $u \in [0, 2\pi)$, with v in $v = 5\tanh(0.5x)$ mm ($x \in [-\pi, \pi)$) and $c = \{25, 40, 50, 75, 100\}$ mm.

These specific parameters for the ideal geometries are chosen to obtain shapes at a wide range of sizes. The same algorithm as for the aortas is used to compute the per-vertex shape operators.

Other Shape Metrics

Fig F analyzes previous shape metrics from the literature. In addition to quantifying rupture risk using Gaussian curvature, investigators predicting aneurysmal rupture risk have utilized other geometric indices, including the L2-norm of the Gaussian curvature $\left(GLN = \frac{1}{4\pi}\sqrt{\sum a_m \sum \kappa_{\mathrm{g}_m}^2 a_m}\right)$, area-averaged Gaussian curvature $\left(GAA = \sum \kappa_{\mathrm{g}_m} a_m / \sum a_m\right)$, area-averaged mean curvature $(MAA = \sum \kappa_{\mathrm{m}_m} a_m / \sum a_m)$, and L2-norm of the mean curvature $\left(MLN = \frac{1}{4\pi}\sqrt{\sum \kappa_{\mathrm{m}_m}^2 a_m}\right)$ [10–12]. While one study concluded that the GAA is highly correlated with wall stress [13], another concluded that the GLN and MLN best classified unruptured vs. ruptured AAAs [14].

The drawbacks of these measures become evident when applied to the patient set in Fig F Panel I demonstrates how the GLN is essentially a reparameterized version of A_T, as it clearly scales with size, with the one term being mathematically equivalent to A_T (Ib) and the second term demonstrating no correlation with the data (Ic). The GAA (panel II) again scales with size, and the MAA and MLN are poor classifiers of aortic

disease state (panels III and IV). Panels V and VI test shape measures from the broader biological literature that examine the scaling of areas and volumes: the flatness index $\gamma = A^3/V^2$ and the sphericity index $\chi = \frac{4.836V^{2/3}}{A}$ [15]. The sphericity index is defined as unity for a sphere such that $0 < \chi < 1$, making it a bounded form of the flatness index. The two measures are related by $\gamma \propto \chi^{-3}$. As shown, both measures simply scale with size. Neither provides a constant baseline for non-pathologic patients.

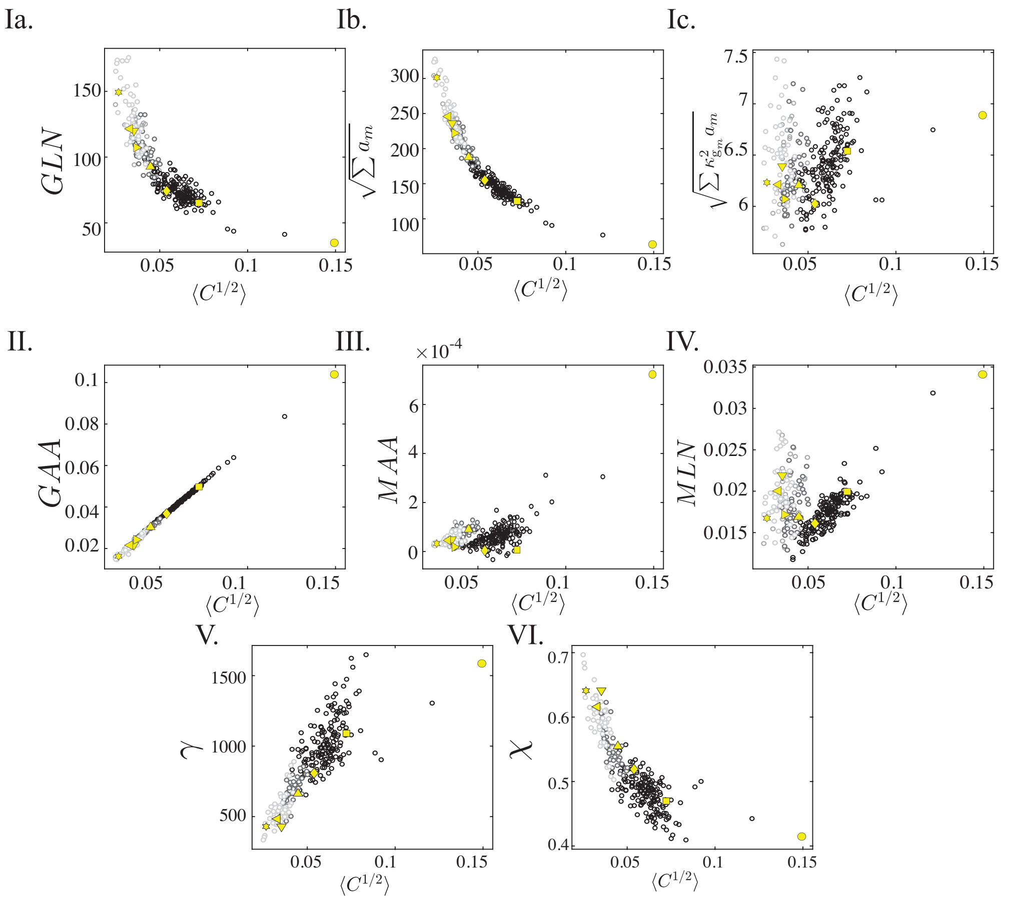

Fig F. Various Shape Literature Shape Metrics Shape metrics from the literature. Ia. The GLN distinguishes the patient groups, but it scales with $\langle C^{1/2} \rangle$, as can be seen from its increasing value with increasing size of the non-pathologic aortas (while δK remains constant throughout this regime). The GLN is mathematically composed of two parts. Ib. The first term is simply the sum of the individual element areas, which is the total aortic area. Ic. The second term $\sqrt{\sum \kappa_{Gm}^2 a_m}$ carries no information, as evidenced by the absence of a trend. II. The GAA is a size metric as it linearly scales with $\langle C^{1/2} \rangle$. III. The MAA carries no meaningful information, as it cannot separate normal from diseased aortas. IV. The MLN similarly does not correlate with aortic disease state. V. The flatness index $\left(\gamma = A^3/V^2\right)$ scales with size and is non-constant for non-pathologic aortas of different sizes. VI. The sphericity index $\left(\chi = \frac{4.836V^{2/3}}{A}\right)$ also scales with size.

Finite Element Simulations

A sphere is defined with inner radius 3 mm and thickness 0.1 mm (see Fig 11 in the main paper). A linear tetrahedral mesh (C3D4 elements) is applied with a target of 5 elements through the thickness for a total of 214,643 elements. Note that linear tetrahedral elements were also employed in multiple works on computational modeling with complex patient-specific geometry [16, 17], this mesh type is selected to be able to extend this work in the future with patient-specific data. A neo-Hookean material model is used with material parameters $c_{10} = \frac{\mu}{2} = 0.005$ MPa and $D = 2$ (1/MPa) (μ is the shear modulus and $K = 2/D = 1$ MPa is the bulk modulus). The inner surface is pressurized to 30 mmHg. During the pressurization process, no growth is prescribed to the aortic wall. This is followed by growth in randomly selected surface partitions at a rate of $\dot{\nu} = 0.008$ m/s. Pressurization is performed for 10 ms and growth for 50 ms for 60 ms total in the simulation. No boundary conditions are applied. Eighty frames are selected from the simulation. To obtain the consistent orientation of normal vectors, the outer surface is isolated. The surface is smoothed to reduce simulation noise, and the geometry is re-meshed in ScanIP to apply an optimal mesh for calculating discrete derivatives.

To demonstrate the loss in utility for Gaussian curvature when global size significantly changes, pressurization and growth are modeled in an idealized aortic geometry (see Fig 12 in the main paper). An ideal aorta is defined in the shape of a candy cane consisting of a cylinder of length 140 mm attached to a half-torus with a cross-sectional radius of 14 mm and a toroidal radius of 28 mm. A linear tetrahedral mesh with a target of 5 elements through the thickness for a total of 239,777 elements is used. The ideal aorta is simulated using the same material property as the sphere but with slightly different pressurization and growth parameters. The simulation applies 225 mmHg of pressure in 40 ms followed by 60 ms of growth in randomly selected surface partitions at a rate of $\dot{\nu} = 0.018$ m/s., resulting in 100 ms of simulation time in total. The surface partitions are manually selected to be roughly evenly distributed along the surface. From the simulation, 100 frames are selected. The outer surface is extracted to obtain a consistent orientation of normal vectors. Then, the geometry is smoothed to reduce simulation noise, and it is re-meshed in ScanIP to apply an optimal mesh for calculating discrete derivatives.

Analysis on Pre-Operative Data

A critical component of the proposed disease state progression trend is the predictive capabilities a singular patient's preoperative aortic condition can have on the success of an impending TEVAR intervention. Fig G shows that for only the last preoperative scan per patient, an analogous trend amongst the normal/diseased and successful/unsuccessful sub-cohorts is still observed. This result suggests that a singular preoperative scan per patient may contain the predictive information required to accurately classify the success of TEVAR-eligible patients. Furthermore, the preoperative state of the aorta in one instance may provide information about its trajectory through this space, potentially introducing a model that may suggest the timeliness of intervention.

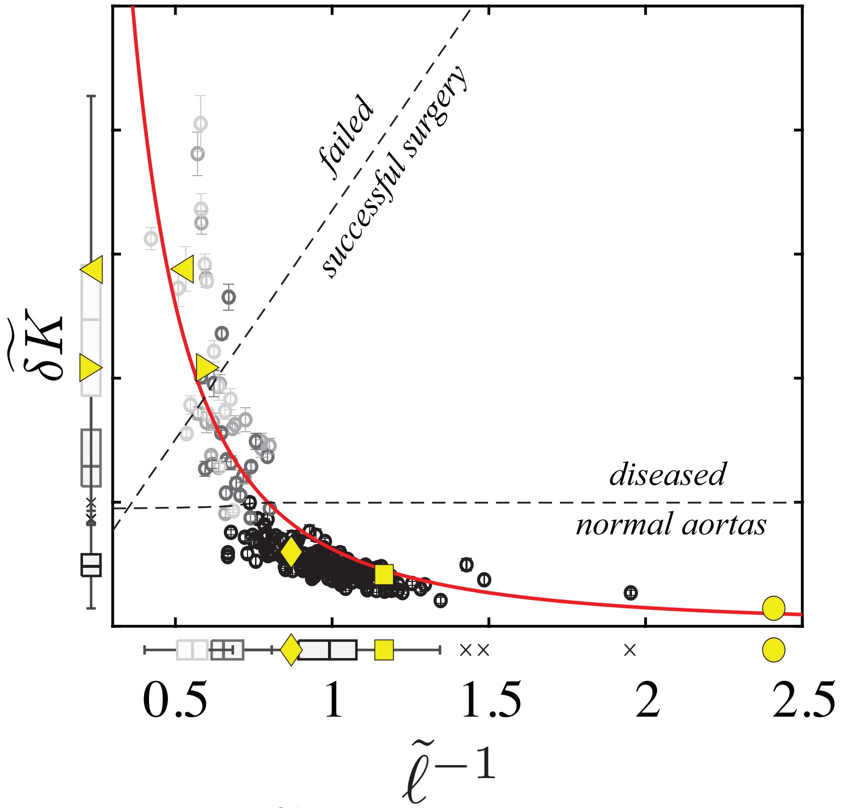

Fig G. Aortic Clustering in $(\widetilde{\delta K}, \tilde{\ell}^{-1})$-space for Pre-Operative Data
The optimal two-dimensional aortic geometric feature space with independent axes for size and shape using only the last preoperative scan per patient. Analogous to Fig 7 III in the main paper, the solid red curve $\widetilde{\delta K} = 1.2\tilde{\ell}^{-2}$ is the best fit to the data. The aortas separate into shape-invariant (normal) and shape-fluctuating (diseased) populations.

Reference Table of Main Variables

A_T	total aortic area
C_i	per-vertex Casorati curvature
$\langle C^{1/2} \rangle$	mean Casorati curvature
$\langle C^{1/2} \rangle^{-1}$	inverse mean Casorati curvature
K	total curvature
$\langle K \rangle$	mean total curvature
δK	fluctuation in total curvature
$\widetilde{\delta K}$	normalized δK
$\sum K$	sum of per-partition total curvatures
k_1	first principal curvature
k_2	second principal curvature
k_{1i}	per-vertex first principal curvature
k_{2i}	per-vertex second principal curvature
κ_m	mean curvature
κ_g	Gaussian curvature
$\overline{\kappa}_{\mathrm{g}j}$	mean Gaussian curvature inside of a partition
$\delta\kappa_{\mathrm{g}}$	fluctuation in Gaussian curvature across the manifold
ℓ	aortic inner scale
ℓ^{-1}	inverse of ℓ
$\tilde{\ell}^{-1}$	ℓ^{-1} normalized to the mean ℓ of the normal aortas
$\mathcal{L}$	centerline length
R_m	maximum aortic radius
$2R_m$	maximum aortic diameter
$\langle R \rangle$	mean aortic radius
$\widetilde{R_2}$	median aortic radius
$\mathbb{S}_i$	per-vertex shape operator
$\mathcal{S}$	external aortic shape
$\mathbf{S}^3$	unit sphere
T^2	two-dimensional torus
V	total aortic volume

Fig H. Reference Table of Main Variables
Pre-operative TBAD scans, as well as all non-pathologic scans, are plotted in the geometric feature space, with independent axes for size and shape.

References

1. Rusinkiewicz S. Estimating curvatures and their derivatives on triangle meshes. In: Proceedings. 2nd International Symposium on 3D Data Processing, Visualization and Transmission, 2004. 3DPVT 2004.; 2004. p. 486-93. Available from: https://ieeexplore.ieee.org/document/1335277.

2. Ben Shabat Y, Fischer A. Design of Porous Micro-Structures Using Curvature Analysis for Additive-Manufacturing. Procedia CIRP. 2015 Jan;36:279-84. Available from: https://www.sciencedirect.com/science/article/pii/S221282711500431X.

3. Max N. Weights for Computing Vertex Normals from Facet Normals. Journal of Graphics Tools. 1999 Jan;4(2):1-6. Publisher: Taylor & Francis _eprint: https://doi.org/10.1080/10867651.1999.10487501. Available from: https://doi.org/10.1080/10867651.1999.10487501.

4. Lin J. Divergence measures based on the Shannon entropy. IEEE Transactions on Information Theory. 1991 Jan;37(1):145-51. Conference Name: IEEE Transactions on Information Theory. Available from: https://ieeexplore.ieee.org/document/61115.

5. Dalton JE, Benish WA, Krieger NI. An Information-Theoretic Measure for Balance Assessment in Comparative Clinical Studies. Entropy. 2020 Feb;22(2):218. Number: 2 Publisher: Multidisciplinary Digital Publishing Institute. Available from: https://www.mdpi.com/1099-4300/22/2/218.

6. Tsigalou C, Panopoulou M, Papadopoulos C, Karvelas A, Tsairidis D, Anagnostopoulos K. Estimation of low-density lipoprotein cholesterol by machine learning methods. Clinica Chimica Acta. 2021 Jun;517:108-16. Available from: https://www.sciencedirect.com/science/article/pii/S000989812100070X.

7. Freedman D, Diaconis P. On the histogram as a density estimator:L2 theory. Zeitschrift für Wahrscheinlichkeitstheorie und Verwandte Gebiete. 1981 Dec;57(4):453-76. Available from: https://doi.org/10.1007/BF01025868.

8. Lo SH, Lau TS. Mesh generation over curved surfaces with explicit control on discretization error. Engineering Computations. 1998 May;15(3):357-73. Available from: https://www.emerald.com/insight/content/doi/10.1108/02644409810208516/full/html.

9. Spivak M. A Comprehensive Introduction to Differential Geometry, Vol. 2, 3rd Edition. 3rd ed. Houston, Tex: Publish or Perish; 1999.

10. Martufi G, Di Martino ES, Amon CH, Muluk SC, Finol EA. Three-dimensional geometrical characterization of abdominal aortic aneurysms: image-based wall thickness distribution. Journal of Biomechanical Engineering. 2009 Jun;131(6):061015. Available from: https://doi.org/10.1115/1.3127256.

11. Shum J, Xu A, Chatnuntawech I, Finol EA. A Framework for the Automatic Generation of Surface Topologies for Abdominal Aortic Aneurysm Models. Annals of Biomedical Engineering. 2011 Jan;39(1):249-59. Available from: https://doi.org/10.1007/s10439-010-0165-5.

12. Ma B, Harbaugh RE, Raghavan ML. Three-Dimensional Geometrical Characterization of Cerebral Aneurysms. Annals of Biomedical Engineering. 2004 Feb;32(2):264-73. Available from: http://journals.kluweronline.com/article.asp?PIPS=479330.

13. Chauhan SS, Gutierrez CA, Thirugnanasambandam M, De Oliveira V, Muluk SC, Eskandari MK, et al. The Association between Geometry and Wall Stress in Emergently Repaired Abdominal Aortic Aneurysms. Annals of biomedical engineering. 2017 Aug;45(8):1908-16. Available from: https://www.ncbi.nlm.nih.gov/pmc/articles/PMC5529246/.

14. Lee K, Zhu J, Shum J, Zhang Y, Muluk SC, Chandra A, et al. Surface Curvature as a Classifier of Abdominal Aortic Aneurysms: A Comparative Analysis. Annals of Biomedical Engineering. 2013 Mar;41(3):562-76. Available from: https://doi.org/10.1007/s10439-012-0691-4.

15. Adam JA. Mathematics in Nature: Modeling Patterns in the Natural World. Princeton University Press; 2003. Available from: https://www.jstor.org/stable/j.ctt7rkcn.

16. Baillargeon B, Rebelo N, Fox DD, Taylor RL, Kuhl E. The Living Heart Project: A robust and integrative simulator for human heart function. European Journal of Mechanics - A/Solids. 2014 Nov;48:38-47. Available from: https://www.sciencedirect.com/science/article/pii/S0997753814000564.

17. Weickenmeier J, Kuhl E, Goriely A. Multiphysics of Prionlike Diseases: Progression and Atrophy. Physical Review Letters. 2018 Oct;121(15):158101. Publisher: American Physical Society. Available from: https://link.aps.org/doi/10.1103/PhysRevLett.121.158101.
