# Supplementary material for: The geometric evolution of aortic dissections: Predicting surgical success using fluctuations in integrated Gaussian curvature
Source: PLoS Comput Biol. 2024 Feb 2;20(2):e1011815. doi: 10.1371/journal.pcbi.1011815 (PMC10866512; doi:10.1371/journal.pcbi.1011815)
Supplement: Attachment — Submitted filename: PCOMPBIOL-D-23-01525_ReviewerComments.docx [file pcbi.1011815.s003.docx]

The authors present a novel metric, total curvature (𝜹Κ), which is able to stratify aortic geometries in a way that is not affected by the size of the aorta in comparison to maximum diameter. Their results demonstrate that this metric could potentially predict outcomes for patients with diseased aortas, influencing treatment decisions. However, age does not seem to be considered in their analysis (aside from mentioning normal growth of 0.5 mm/year in the intro). A large mean age gap (37 vs 63) is present between controls and patients who received TEVAR. Because age affects vessel elasticity and curvature, the significance of their findings may be inflated between control and TEVAR. Therefore, without this consideration for age, it is hard to know the full impact of this work. Once age is considered, I believe this work will be useful to the field with the potential to make a positive clinical impact.

Major Revisions:

1. As stated, I believe age needs to be considered to understand the true impact of this work. Below I’ve listed some ideas that would be helpful to see incorporated into the manuscript
   a. In the demographics table of TEVAR patients, split the table into two: successful TEVAR, unsuccessful TEVAR. If the mean ages between groups are similar, this will help make the results more convincing
   b. Make sub-cohorts of age-matched successful, unsuccessful, and control subjects and plot 𝜹Κ
   c. Make a new plot of 𝜹Κ vs age for each aorta to visualize how age can affect total curvature
   d. In the discussion, make sure to not overstate the findings of this study. An example being “normal size-related development and pathologic shape-related changes” as age can cause shape change but would not be considered pathologic. Again, “we show that aortas with high 𝜹Κ independently classify as high risk for clinical complication” is a big statement to make without adjusting for age-related shape changes.
2. Another concern with the data is that the actual n sizes are 18 and 18 (for TEVAR groups) and 93 controls. Please provide justification for these sample sizes including a power analysis.
3. A related concern is that each CTA from one subject is being considered as a separate aorta in the analyses. Please justify this decision and consider creating a plot of the data using one CTA per subject.
4. More information is needed about the segmentation process. Were all segmentations conducted by the same person? Was the outer or inner wall segmented from the CTAs? If it was the inner wall, in the TEVAR cases, there likely will be increased curvature due to the stent structure (i.e., the pattern of the stent is often visible in the segmentation), which would further separate TEVAR cases from controls. Based on this concern, plotting the data using only pre-TEVAR data would eliminate that possible issue.
5. Plotting only pre-TEVAR would also provide helpful insight for clinical translation of this work, as a difference in pre-TEVAR curvature between successful and non-successful TEVAR subjects would suggest that 𝜹Κ could be used to predict surgical outcomes in TEVAR-eligible patients. In the other plots, please justify the reason to include pre- and post-TEVAR in the same group.

Minor revisions:

1. The introduction of the manuscript reads almost like a textbook or review paper. Please condense to the relevant information as that will help the reader understand the focus of this paper. I would suggest condensing from 3.5 pages to about 1.5. In addition, I would keep the focus more on the curvature (Κ) measure.
2. The variables in this manuscript are difficult to keep track of. I think it would be helpful for the readers to create a table summarizing the main variables and their meanings so it can be quickly referenced as I found myself having to dig back through the text to find remind myself what a variable meant. Sometimes, I was never able to clearly find it so please make sure that all variables are explicitly defined (example is T^2^).
3. In the plots or results, I think it would be helpful to denote the TAA patients or include any differences found between TAA and TBAD groups as I would imagine the surface curvature of a false lumen may be greater than the surface curvature of a TAA in the same region since the wall is thinner.
4. In the first paragraph of the methods which states the three subject subgroups, I would state the n (i.e., 18, 18, 93) as the n for controls is only included in the appendix table.
5. Also in the first paragraph of methods, I think it is important to state where these images are coming from and relevant details (e.g., hospital, registry, scanner type, etc.).
6. In the “Meshing Algorithm …” section, the five step process includes noise reduction but is not shown in Figure 2. It would be helpful to include an example of that.
7. In the same section, please clarify the sensitivity analysis process. Normally in CFD, the sensitivity analysis is done on one or two cases and then an “optimal” mesh size is chosen. Here, the text seems to say that 15 mesh surfaces are generated for each aorta. Please further explain why 15 meshes are created instead of determining an optimal mesh size from one or a few cases so that one mesh can be used for each aorta.
8. Equation 9 should be “S_e_” instead of “S” as defined in the sentence below.
9. In section 3.2, the first sentence has a typo: dat should be data

Figure revisions:

1. All figures: make sure all variables are defined in the captions.
2. Figure 1: There are undefined yellow symbols. I think it would also be helpful to include the age of each aorta beneath it. Also, the arrow above t makes it seem like the aortas are increasing in age, but I think it’s supposed to be increasing in total curvature? So consider re-labeling the arrow.
3. Figure 2: It would be helpful to have a zoomed image of the meshed geometry, displaying the element shapes
4. Figure 7: There is a discrepancy in Figure 7 and Figure 1 where the descriptions say one-year-old and three-year-old. Please verify the age and correct one of them.
5. Figure 7: It would be helpful to outline the yellow representative case symbols with their appropriate group (black, light gray, dark gray)
6. Figure 9: The plot switches groups from grayscale to multi-colored. Please keep it consistent or if I’m missing something, please explain why the change is made. On a related note, I think changing the shape between sub-cohorts would be help visualization in addition to the 3 colors.
7. Figure 9: label each plot with what parameter(s) is being assessed. Also plot Ic and IIb have solid lines instead of dotted, but I’m not sure of the reason for this.
8. Figure 12: In caption, explain what the x-axis variable means. Also in 12II it looks like the representative case on the far right is smaller in the colored version which makes me wonder about the scale of each representative case shown. Please make it consistent and consider adding a scale bar.
