# Supplementary material for: The geometric evolution of aortic dissections: Predicting surgical success using fluctuations in integrated Gaussian curvature
Source: PLoS Comput Biol. 2024 Feb 2;20(2):e1011815. doi: 10.1371/journal.pcbi.1011815 (PMC10866512; doi:10.1371/journal.pcbi.1011815)
Supplement: Attachment — Submitted filename: Reviewer_Comments_Responses.docx [file pcbi.1011815.s004.docx]

We thank the reviewers on their careful reading of our manuscript. The comments were very helpful, and we have made a strong effort to address nearly all of them thereby substantially strengthening the paper. Below is a detailed response to each of the comments. We believe this paper is now much stronger and hope the editor and reviewers agree with moving forward publishing our novel approach to classifying aortic shape.

**REVIEWER 1**

In this work, methods of topology are applied to study aortic morphogenesis throughout the life-cycle of an aorta. The authors work with aortic surfaces derived from cross-sectional images from CT scans from both normal patients and patients with aortic dissections at various stages of disease (302 aortas in total). From this inspection, two clinical regimes are observed: shape preserving (normal) growth and (diseased degeneration) growth with shape changes.

This leads the authors to define a dual parameterization space of both aortic size and shape (two orthogonal metrics) by using the total curvature K as the primary measure of shape. It is first proved that all aortas scale as generalized bent cylinders parameterizable by a single length scale. It is then shown that the variance of K captures the shape evolution by characterizing local shape changes.

It is finally shown that the consideration of these combined metrics outperform other size and shape measures from the clinical and engineering literatures when applied to the predictive classification of Thoracic Endovascular Aortic Repair (TEVAR) success. It is suggested that such a classification space can be clinically applied to pre-operative treatment planning for aortic dissection patients.

The work done is original and innovative, providing relevant methods and results to researchers in the field. The manuscript could be considered for publication.

We thank the reviewer for his positive comments on the originality and innovativeness of our work.

The following remarks should be addressed by the authors:

P5: "We prove that all aortic shapes are homeomorphic to T^2". Please define T^2, which is mentioned several times in the manuscript. This has now been defined with all other variables and symbols used in the paper in a new table (figure 20).

P5: "Type IA endoleak". Do you mean 1A, which is defined below in the text? Please define here. Correct, type 1A (proximal seal zone) endoleak. The notation has been changed.

P6: "Isolation of the outer surface". What about the inner surface? Is the thickness variation/evolution not taken into account? Would the inner surface lead to the same conclusions? The initial segmentation mask is constructed from the lumen of the aorta, followed by selection of the outer wall of the solid mask which corresponds to the inner surface of the aorta. This method of segmentation is standard for aortic surfaces obtained using contrast enhanced CT imaging. The inner surface is the most robust method of selecting aortic surfaces, given the difficulty in correctly identifying the outer aortic wall on CTA. There is no consistent clinical imaging modality that allows reliable measurement of aortic thickness throughout the length of the aorta. The following sentence was added on page 18 to clarify this: “The outer edge of the segmentation mask corresponds to the aortic inner wall, which is the most robust method of selecting the aortic surface given the difficulty in correctly delineating the outer aortic wall on CTA.”

P10. Results: Why are major/minor results (3.1)-(3.5) introduced in the order 1,2,4,3,5 and never referred to below (in that order or any other)? This comment was addressed in the paper.

P10. FE simulations: - "All parameters are determined from Fe instead of the total deformation F in the case growth is triggered." Why Fe? It is F, not Fe, what determines the observable geometry. The section on Finite element simulations has been modified to address this comment. In particular the following sentence was added: “Adopting the assumption that Fg does not explicitly contribute to the free energy in previous work on computational modeling of multiplicative growth [54–56], a neo-Hookean (NH) strain energy function of the following form is used: W = W(F,Fg) = We(Fe).”

- Se in Eq. (10) is defined as the second Piola-Kirchhoff stress, but how it is obtained is not defined anywhere. Indeed, the actual second Piola-Kirchhoff stress is that given in Eq. (9), i.e. S. The section on Finite element simulations has been modified to address this comment, the notation has been corrected. Our implementation of the growth follows the standard decomposition approach well documented in references 54-56.

- Why is isotropic growth with constant growth rate in the longitudinal-circumferential plane assumed? Why is the tissue not allowed to grow in the through-the-thickness direction? This is somehow related to my previous comment on P6. The simulations are performed with the intent of understanding the source of the $\delta K$ divergence seen in diseased aortas using a controllable set of geometries. There is little clinical data available to support that thickness changes occur to the same level as changes in area in aneurysmal degeneration of aortic dissections. While some aortas may indeed thicken with dilation others likely thin, and most probably remain unchanged. In my own hands (Dr. Luka Pocivavsek), having performed over 100 open aortic operations for aneurysms, I would not say any pattern of consistent change in thickness could be noted, while changes in surface area are very clear. As such, in an attempt to mimic the clinical data, only in-plane growth was implemented.

Fig. 12: "Ib. dK captures the increasing surface degeneration due to growth. Ic. dAj does not capture this degeneration." It seems that dAj follows the same tendency as dK in Fig. 12. Please explain better these conclusions. The caption to figure 12 was updated to point out while there is a similar trend in the area scaling, the error bars are significantly larger with area fluctuations. Furthermore, the yellow symbols (which can be used as a visual guide for the different geometries) are far more compressed in the area axis than along the $\delta K$ axis.

P23 FE simulations: Parameters used for the NH model give as a result a nearly incompressible behavior. The type of elements used are not specified. Please specify them and clarify if they prevent possible volumetric locking. We have added the element type (C3D4) in the Appendix section for FEM. We used explicit solver so some amount of compressibility is introduced and we also correct a typo of the bulk modulus. We used K=2/D which results in the ratio of K/mu=100 which is within the limit that Abaqus suggests to incorporate some amount of compressibility while reducing noise in the dynamic solution. We stick with tet element because we want to use this approach in the future with patient specific modeling and we don’t observe volumetric locking. We also refer to the use of linear tetrahedral elements in other growth modeling work with patient specific model [ref 1,2].

Ref 1: “The living heart project: a robust and integrative simulator for human heart function” Eur. J. Mech. A solids 2014, 48: 38-47

Ref 2: “The multiphysics of prion-like diseases: Progression and atrophy” Physical Review Letters 2018, 121: 158101

**REVIEWER 2**

The authors present a novel metric, total curvature (𝜹Κ), which is able to stratify aortic geometries in a way that is not affected by the size of the aorta in comparison to maximum diameter. Their results demonstrate that this metric could potentially predict outcomes for patients with diseased aortas, influencing treatment decisions. However, age does not seem to be considered in their analysis (aside from mentioning normal growth of 0.5 mm/year in the intro). A large mean age gap (37 vs 63) is present between controls and patients who received TEVAR. Because age affects vessel elasticity and curvature, the significance of their findings may be inflated between control and TEVAR. Therefore, without this consideration for age, it is hard to know the full impact of this work. Once age is considered, I believe this work will be useful to the field with the potential to make a positive clinical impact.

We thank the reviewer for their comments and are happy to read that they believe our work can make a “positive clinical impact”. The reviewer makes a good point about the difference in age between the normal cohort and the dissection cohort. In 1a, we outline that in the dissection cohort, the two TEVAR groups are well matched in age. The low mean in the normal aortas is driven by the fact that most of these patients are trauma patients who received CT pan scans. Given that trauma patients on the Southside of Chicago tend to be younger, this group has a lower mean (37) but wide SD of 18 with a range of 1 to 86. We believe it is beyond the scope of this paper to fully explore the normal patient population, however this is an ongoing project in our group with a full separate manuscript under preparation. As seen in figures 7 and 9 the change in shape as measured by $\delta K$ is very weak throughout the entire control cohort. We find no statistical difference in shape as measured using $\delta K$ between normal patients under 65 and those older than 65. Because this analysis is part of a separate paper on the normal cohort, and the current paper is focused on dissection patient classification, we believe the nearly constant $\delta K$ shown in our current data for the entire normal patient population is sufficient to support this point. We now address the specific points below:

Major Revisions:

1. As stated, I believe age needs to be considered to understand the true impact of this work. Below I’ve listed some ideas that would be helpful to see incorporated into the manuscript
   a. In the demographics table of TEVAR patients, split the table into two: successful TEVAR, unsuccessful TEVAR. If the mean ages between groups are similar, this will help make the results more convincing The mean ages between groups are statistically similar: mean of 59.06 years for successful TEVAR with standard deviation of 10.88 years vs. mean of 67.44 years for failed TEVAR with standard deviation of 15.52 years. This gives a two-tailed P value of 0.08, as such by conventional criteria (<0.05) the age difference between the two TEVAR populations is not statistically significant. The similar means, coupled with the broad age distribution, addresses this comment. We do not explicitly study age since TBAD is mostly a disease of older patients with cardiovascular risk factors. We explicitly did NOT include patients with connective tissue disorders who tend to present with dissections at young ages, since TEVAR is contra-indicated in aortopathy. This is indicated in Table (figure 14) where Known Marfan is 0 for both groups.
   b. Make sub-cohorts of age-matched successful, unsuccessful, and control subjects and plot 𝜹Κ Since the age difference between the two TEVAR groups is not significant, such sub-cohorts of age-matched groups would not be meaningful. In the future, as we continue to expand this methodology and integrate more patients including much younger dissection patients with possible aortopathy such analysis can be undertaken.
   c. Make a new plot of 𝜹Κ vs age for each aorta to visualize how age can affect total curvature See above
   d. In the discussion, make sure to not overstate the findings of this study. An example being “normal size-related development and pathologic shape-related changes” as age can cause shape change but would not be considered pathologic. Again, “we show that aortas with high 𝜹Κ independently classify as high risk for clinical complication” is a big statement to make without adjusting for age-related shape changes. These statements were modified in the paper. We have clarified that pathologic changes related to dissection (see page 16).
2. Another concern with the data is that the actual n sizes are 18 and 18 (for TEVAR groups) and 93 controls. Please provide justification for these sample sizes including a power analysis. We acknowledge that this is a limitation of the study, the 36 TEVAR patients in the UofC cohort are real-world patients. Because the study is limited to patients with TBAD with dissection distal to LSA, we needed to exclude any patient with type A and residual TBAD. Furthermore, we were only interested in studying patients that underwent standard zone 2/3 TEVAR without distal extension into the peri-visceral aorta. While these numbers are small, they are comparable with most aortic clinical trials: Gore 08-01 NCT 00908388 Trial N=46, Gore 08-02 (NCT00874250) Trail N=18, Medtronic Dissection trial (NCT01114724) N=30, and Endospan European Arch dissection trial N=25. Given the comparable size with well-established clinical trials, we believe the size of our cohort will not be a limitation in the clinical acceptance of our analysis.
3. A related concern is that each CTA from one subject is being considered as a separate aorta in the analyses. Please justify this decision and consider creating a plot of the data using one CTA per subject. We have added the following new paragraph in the appendix on page 25:

“Analysis on Pre-Operative Data: A critical component of the proposed disease state progression trend is the predictive capabilities a singular patient’s preoperative aortic condition can have on the success of an impending TEVAR intervention. Figure 19 shows that for only the last preoperative scan per patient, an analogous trend amongst the normal/diseased and successful/unsuccessful sub-cohorts is still observed. This result suggests that a singular preoperative scan per patient may contain the predictive information required to accurately classify the success of TEVAR-eligible patients. Furthermore, the preoperative state of the aorta in one instance may provide information about its trajectory through this space potentially introducing a model which suggests the timeliness of intervention. “

A new figure (19) is also included, where we only plot the last-pre operative point. The equivalency of the data in this figure and figure 7III shows that our analysis and the separation in patient groups holds even if only the pre-operative CT scans are used per patient.

4. More information is needed about the segmentation process. Were all segmentations conducted by the same person? Was the outer or inner wall segmented from the CTAs? If it was the inner wall, in the TEVAR cases, there likely will be increased curvature due to the stent structure (i.e., the pattern of the stent is often visible in the segmentation), which would further separate TEVAR cases from controls. Based on this concern, plotting the data using only pre-TEVAR data would eliminate that possible issue. All segmentations for diseased CTAs were conducted by the same person. The segmentations for the non-pathologic scans were conducted by another person, but the same segmentation protocol was used. The initial segmentation mask is constructed from the lumen of the aorta, followed by selection of the outer wall of the solid mask which corresponds to the inner surface of the actual aorta. The segmentation was manually and carefully corrected to remove the shape effect of the stent structure for the TEVAR cases. Furthermore, the inner surface is the most robust method of selecting aortic surfaces, given the difficulty in correctly identifying the outer aortic wall on CTA. In addition, the pre-TEVAR data addresses this issue. On page 18, the following sentence was added to clarify the outer/inner wall question: “The outer edge of the segmentation mask corresponds to the aortic inner wall, which is the most robust method of selecting the aortic surface given the difficulty in correctly delineating the outer aortic wall on CTA. “
5. Plotting only pre-TEVAR would also provide helpful insight for clinical translation of this work, as a difference in pre-TEVAR curvature between successful and non-successful TEVAR subjects would suggest that 𝜹Κ could be used to predict surgical outcomes in TEVAR-eligible patients. In the other plots, please justify the reason to include pre- and post-TEVAR in the same group.

In the main text both preoperative and postoperative scans populate the figures and analysis to study the instantaneous *aortic state* as it pertains the underlying trend described by aortic shape. In future studies with larger assortments of data, a more thorough analysis can be performed with the statistical significance to assess the predictive capabilities of preoperative-exclusive imagining data. However, a new section has been added to the appendix which reperforms the size-shape plotting analysis using only the last preoperative scan per patient in the dataset. (see response to comment 3)

Minor revisions:

1. The introduction of the manuscript reads almost like a textbook or review paper. Please condense to the relevant information as that will help the reader understand the focus of this paper. I would suggest condensing from 3.5 pages to about 1.5. In addition, I would keep the focus more on the curvature (Κ) measure. This is a paper intended for multiple audiences with multiple types of expertise from clinicians to computer scientist and as such requires a comprehensive introduction. The problem of aortic classification carries much weight since decisions to intervene on the aorta are not low-stakes decisions. We believe a careful exposition of the size-based measure is critical. Moreover, the known open questions about shape clinically need to be addressed. The problem of defining aortic shape has been tackled before, however without much success. We believe the introduction can be skimmed by those with familiarity with the subjects otherwise it provides a valuable survey of the problem for those who may not know it. Given that PLOS Computational Biology does not have a page limit, simply reducing it for the sake of size is not necessary.
2. The variables in this manuscript are difficult to keep track of. I think it would be helpful for the readers to create a table summarizing the main variables which will go into appendix and their meanings so it can be quickly referenced as I found myself having to dig back through the text to find remind myself what a variable meant. Sometimes, I was never able to clearly find it so please make sure that all variables are explicitly defined (example is T^2^). We have added table/figure 20 to the appendix which defines all the mathematical variables.
3. In the plots or results, I think it would be helpful to denote the TAA patients or include any differences found between TAA and TBAD groups as I would imagine the surface curvature of a false lumen may be greater than the surface curvature of a TAA in the same region since the wall is thinner. There was only one TAA patient and that patient was **not** included in the clinical classification cohort (and thus not included in the demographics table, which is only for that cohort).
4. In the first paragraph of the methods which states the three subject subgroups, I would state the n (i.e., 18, 18, 93) as the n for controls is only included in the appendix table. The data is updated in the appendix demographic tables.
5. Also in the first paragraph of methods, I think it is important to state where these images are coming from and relevant details (e.g., hospital, registry, scanner type, etc.). We added the following paragraph on page 5: “Computed tomography images were obtained via the Human Imaging Research Office (HIRO) at the University of Chicago Medicine, an institutional imaging research core that provided HIPAA-compliant, deidentified DICOM data for patients requested for this study. A variety of scanners were used to collect radiographic information.”
6. In the “Meshing Algorithm …” section, the five step process includes noise reduction but is not shown in Figure 2. It would be helpful to include an example of that. Figure 2 was updated.
7. In the same section, please clarify the sensitivity analysis process. Normally in CFD, the sensitivity analysis is done on one or two cases and then an “optimal” mesh size is chosen. Here, the text seems to say that 15 mesh surfaces are generated for each aorta. Please further explain why 15 meshes are created instead of determining an optimal mesh size from one or a few cases so that one mesh can be used for each aorta. The caption for figure 2 was updated to clarify this point. An optimal mesh size cannot be determined since geometrically, a range of different mesh sizes and smoothing parameters generate plausible outcomes. We identified a meshing strategy which gave consistent and invariant results. The data in figures 7, 11, and 12 have error bars which show the spread of the data points based upon the different degrees of smoothing, meshing density, and partitioning. This is indicated in the methods.
8. Equation 9 should be “S_e_” instead of “S” as defined in the sentence below. This comment was addressed.
9. In section 3.2, the first sentence has a typo: dat should be data This comment was addressed.

Figure revisions: These comments were all addressed.

1. All figures: make sure all variables are defined in the captions.
2. Figure 1: There are undefined yellow symbols. I think it would also be helpful to include the age of each aorta beneath it. Also, the arrow above t makes it seem like the aortas are increasing in age, but I think it’s supposed to be increasing in total curvature? So consider re-labeling the arrow. The figure was changed to make clearer that we are not showing a progression of time of a given aorta, but more generally show representative pediatric, normal adult, and diseased adult aortas.
3. Figure 2: It would be helpful to have a zoomed image of the meshed geometry, displaying the element shapes. Figure 2 was updated to include the zoomed geometries and differences in smoothing and mesh density.
4. Figure 7: There is a discrepancy in Figure 7 and Figure 1 where the descriptions say one-year-old and three-year-old. Please verify the age and correct one of them. This was corrected in the caption of figure 7.
5. Figure 7: It would be helpful to outline the yellow representative case symbols with their appropriate group (black, light gray, dark gray) The yellow symbols are projected onto the y-axis in figure 7 III, they all cluster with the corresponding three groups (black, light gray, dark gray).
6. Figure 9: The plot switches groups from grayscale to multi-colored. Please keep it consistent or if I’m missing something, please explain why the change is made. On a related note, I think changing the shape between sub-cohorts would be help visualization in addition to the 3 colors. We changed to using color in this appendix figure because we wanted a simple correlation between the 1D distribution in figure 9 I and the 2D feature space. The main message of figure 9 is that the green and red distributions can NOT be separated in the 1D size space, irrespective what method is used to draw a boundary (this is equivalent to projecting the data onto the x-axis in the 2D shape-size space). The addition of color in this figure is justified in our opinion.
7. Figure 9: label each plot with what parameter(s) is being assessed. Also plot Ic and IIb have solid lines instead of dotted, but I’m not sure of the reason for this. The lines have been updated.
8. Figure 12: In caption, explain what the x-axis variable means. Also in 12II it looks like the representative case on the far right is smaller in the colored version which makes me wonder about the scale of each representative case shown. Please make it consistent and consider adding a scale bar. The figure was updated and the figures are now to scale. The x-axis variable indicates the length scale, defined as the mean Casorati curvature, and this was added to the figure caption.

**REVIEWER 3**

The manuscript introduces a shape-size space for analyzing the development of the dissected aorta.

Strengths:

- Mathematically sound shape-size feature space.
- Profound analysis of various parameters and alternatives to corroborate the results presented.
- Compelling results.

We thank the reviewer for his kind words concerning the deep analysis and mathematical novelty of our approach.

Weaknesses:

- My main concern is the figures presented. When I look at Figures 1, 2, 3, 7 and 15, I have the impression that the aorta is evolving from a "normal length" aorta to a very short (longitudinally) but tortuous or dilated aorta. In other words, it seems to be getting shorter (at least visually) and ends in the descending thoracic aorta (at best). Figure 1 was changed to make clearer that we are not showing a progression of time of a given specific aorta, but more generally show representative pediatric, normal adult, and diseased adult aortas. This figure is not meant to highlight the changes in global geometry and is not meant to precisely preserve all aspect ratios between the different aortas obtained from different patients, though care was taken to preserve the global aspect ratio as much as possible. As such, when we show these surfaces they are meant as visual guides for representative aortic segmentations to allow the reader a better interpretation of our quantitative data.

It appears to become disproportionately thicker than longer when diseased, see figure 1 on the right. The initial segmentation mask is constructed from the lumen of the aorta, followed by selection of the outer wall of the solid mask which corresponds to the inner surface of the aorta. As such thickness never enters the geometry since we work only with surfaces. This method of segmentation is standard for aortic surfaces obtained using contrast enhanced CT imaging. The inner surface is the most robust method of selecting aortic surfaces, given the difficulty in correctly identifying the outer aortic wall on CTA. There is no consistent clinical imaging modality that allows reliable measurement of aortic thickness throughout the length of the aorta. The following sentence was added on page 18 to clarify this: “The outer edge of the segmentation mask corresponds to the aortic inner wall, which is the most robust method of selecting the aortic surface given the difficulty in correctly delineating the outer aortic wall on CTA.”

- Since the surface partitioning patch size seems to be related to the radius, the question is how this should be assessed when comparing/analyzing the development. Aren't patches of different sizes being compared/analyzed? What are the resulting implications?

This was addressed with our Jenson-Shannon divergence (JSD) analysis in the appendix and with Figure 17. We observed a signal loss at coarse and dense patch totals (and corresponding patch sizes). While the data presented in the main text uses a patching scheme which is a function of radius, we also show that this value is tightly distributed around a mean value of k ~ 100.

The manuscript represents a substantial body of work with significant scientific innovations. I believe that my comments can be taken into account in a revision. I, therefore, advocate accepting this manuscript. We sincerely thank the reviewer on this positive assessment of our work.

Remarks:

- eq11: I do not see the growth rate in equation 11c The growth rate comes in with time in the simulation.
- figure 1: the yellow glyphs remain unclear. What is their meaning and what are they representing? The symbols were removed in figure 1 and only kept in figure 7 where they clearly map onto the shape-size feature space.

**Have the authors made all data and (if applicable) computational code underlying the findings in their manuscript fully available?**

A ‘.csv’ file will be provided in the resubmission upload which contains the entire size-shape feature space explored during the analysis. Regularized logistic regression using the SciKit Learn Data Science (https://scikit-learn.org) package was used for the feature selection and machine learning portions of the analysis. The curvature estimation analysis code will be publicly available.

The [PLOS Data policy](https://urldefense.com/v3/__https:/journals.plos.org/ploscompbiol/s/materials-and-software-sharing__;!!MvNZe7V6M35iZPhbgng-hfU!1faDLy4tagPW9Pnie5zP6fe_GSYnEe9BpZ6vqe_pQ4aCDU5FSDvBvWFvadNSgWHWrey-qZeOVdHAJC0TiGKmxWiwDm8$) requires authors to make all data and code underlying the findings described in their manuscript fully available without restriction, with rare exception (please refer to the Data Availability Statement in the manuscript PDF file). The data and code should be provided as part of the manuscript or its supporting information, or deposited to a public repository. For example, in addition to summary statistics, the data points behind means, medians and variance measures should be available. If there are restrictions on publicly sharing data or code —e.g. participant privacy or use of data from a third party—those must be specified.

Reviewer #1: None

Reviewer #2: **No:** The authors state that data and code is available "upon request", which does not seem to meet the PLOS Data policy stating "the data and code should be provided as part of the manuscript or supporting info, or deposited to a public repository".

Reviewer #3: **No:** as stated, available upon request
